# Cardiovascular and brain effects of liraglutide in transthyretin amyloidosis (ATTR) mice models

**DOI:** 10.7150/ijms.112264

**Published:** 2025-07-10

**Authors:** Mengqing Zhang, Zonglin Li, Xiaoling Cai, Fang Lv, Xin Wen, Chengcheng Guo, Chu Lin, Linong Ji

**Affiliations:** Department of Endocrinology and Metabolism, Peking University People's Hospital, Beijing, China.

**Keywords:** ATTRv, liraglutide, brain, heart failure, transthyretin amyloidosis polyneuropathy, transthyretin cardiac amyloidosis

## Abstract

**Aim**: The effects of glucagon-like peptide-1 receptor agonists (GLP-1RAs) in hereditary transthyretin amyloidosis (ATTRv) remain uncertain. This study aims to investigate whether liraglutide interacts with transthyretin protein (TTR) and thereby exerts therapeutic effects for ATTRv.

**Methods**: High throughput screening was conducted to characterize the drug targets of liraglutide, and microscale thermophoresis was used to observe direct binding of liraglutide to TTR. Humanized RBP4/TTR (normal)and RBP4/TTR^Val50Met^ (ATTRv) mice were constructed, and treated with liraglutide (0.3mg/kg/d) or placebo for 28 days. Fasting plasma glucose, intraperitoneal glucose tolerance test (IPGTT), and plasma brain natriuretic peptide (BNP) were measured. Brain and cardiac tissues were processed with western blot, enzyme-linked immunosorbent assay (ELISA), real-time quantitative polymerase chain reaction (PCR), and pathological staining to evaluate the lesion status in corresponding organs.

**Results:** Liraglutide exhibited high affinity and direct combination ability to TTR. In ATTRv mice, liraglutide significantly decreased the contents of TTR protein in brain compared with placebo. However, the cardiovascular prognosis measurements including heart failure (plasma BNP concentrations), cardiac fibrosis (the relative expression levels of *Cola1* and *TGFβ1* in cardiac tissues), and pathological changes (right ventricular collagen percentage, ventricular septum thickness, left ventricular wall thickness, and left ventricular internal diameter) were statistically comparable between mice receiving liraglutide and placebo treatment.

**Conclusion:** Liraglutide could decrease the deposition of TTR in brain tissues, while it did not improve cardiovascular outcomes in ATTRv mice compared to placebo. More researches regarding the mechanisms and therapeutic effects of GLP-1RAs to ATTRv are still required.

## Introduction

Transthyretin (TTR) serves as an important carrying protein for thyroxine (T4) and retinol binding protein 4 (RBP4), which is mainly synthesized in liver. 1,2 Mostly, TTR exists in serum by the form of tetramers. 2 When the structure of TTR monomers is disrupted, the stability of TTR tetramers would severely decline, which promotes the dissociation of TTR tetramers, aggregation of TTR monomers, and subsequent formation of amyloid in tissues, leading to transthyretin amyloidosis (ATTR). 3

ATTR was considered a relative rare disease precedingly. While with the improvements of diagnostic methods, the prevalence of ATTR increased correspondingly. So far, there are two ATTR subtypes namely age-related ATTR (ATTR wild type, ATTRwt) and hereditary transthyretin amyloidosis (ATTR variant, ATTRv). 1 ATTRwt is primarily mediated by aging-related oxidative stress and cells injury, with a median onset age of 70-75 and prevalence more than 25%. 4 For ATTRv, the estimated global prevalence reached 10,186 people (range 5526-38,468), 5 of which more than 150 potential mutation sites have been identified. ATTR comprises a wide range of clinical manifestations including polyneuropathy (ATTR-PN) and cardiomyopathy (ATTR-CM), as well as mixed phenotypes. 6 For ATTRv, of all the mutation sites, p. Val50Met mutation is the most frequent currently, primarily causing a neuropathic phenotype and mixed phenotypes. 7, 8

Glucagon-like peptide-1 receptor agonist (GLP-1RA) represents a novel class of anti-hyperglycemic medication, which exerts glucose lowering effects by stimulating insulin secretion, suppressing glucagon release, inhibiting hepatic gluconeogenesis, and restraining appetite. 9 In addition to its hypoglycemic effect, GLP-1RA also affects central nervous system, reaching the pathological regions of several neurodegenerative diseases especially Alzheimer's disease (AD) or Parkinson's disease (PD), exerting neuroprotective effects. 10 It was demonstrated that liraglutide could introduce neuroprotective effects in patients at high risk of AD, possibly by improving glucose metabolism and functional connectivity in brain cells. 11 Meanwhile, it was also indicated that liraglutide could reduce the load of amyloid-beta plaques in the hippocampus and decrease astrogliosis in the cerebral cortex of mice models with AD. 12 This effect may be mediated through the activation of the phosphoinositol 3-kinase/Akt (PI3K/Akt) pathway, which enhanced the presence of amyloid-beta transporters in cerebrospinal fluid, promoting the and clearance of amyloid-beta. 13

Meanwhile, in respect to the cardiovascular effects of GLP-1RAs, since several cardiovascular outcome trials (CVOTs) have demonstrated that the cardio-protective effects of GLP-1RAs were independent with glycemic control or weight loss 14-16, GLP-1RAs have been proved with specific cardiovascular benefits. 17,18

However, whether GLP-1RAs would contribute to the treatment of neurological and cardiovascular lesions in ATTRv remain unexplored. The aim of this study is to investigate whether liraglutide would treat amyloidosis-related neural and cardiovascular conditions caused by ATTRv, and to further characterize the corresponding underlying mechanisms.

## Materials and methods

### Materials

TTR protein was purchased from sinobiological company (12091-H08H, Beijing, China). Liraglutide was purchased from MedChemExpress company (HY-P0014, New Jersey, USA).

### High throughput targets screening

To validate whether liraglutide would mediate brain and cardiovascular benefits for ATTRv by potentially interacting with TTR or other targets, we implemented high throughput targets screening for liraglutide. The screening platform was Discovery Studio 2017R2 (DS; BIOVIA-Dassault Systemes). The receptor and ligand package generation module within DS was utilized to generate Multi-Target Binding Motifs (MBMS). The optimized form of liraglutide was then linked to its respective potential target through ligand binding, when the ligand score would be subsequently calculated. These computational analyses were performed on an Intel® Xeon® E5-2699 v3 2.30 GHz octadeca-core processor running Windows 8.1 operating system.

### Microscale Thermophoresis

To characterize the direct interaction between liraglutide and TTR, we employed microscale thermophoresis using the Monolith NT.115 instrument (NanoTemper Technologies). Fluorescent labeling of TTR proteins was performed following the manufacturer's protocol.

Different concentrations of liraglutide, ranging from 1.0E-10 M to 1.0E-4 M (10 nM to 10 μM) on a logarithmic scale, were prepared in binding buffer. After incubation, samples were loaded into Monolith NT.115 Series Capillaries (Nanotemper, Germany) and subjected to microthermophoresis using Medium MST-Power and 20% of Excitation-Power. Equilibrium dissociation constant (Kd) values were calculated from repeated measurements using the mass action equation provided by the NanoTemper software. During the experiment, the migration rate of the fluorescently labeled TTR protein in response to a temperature gradient was measured to assess thermophoresis. The migration rate of the labeled TTR is inversely proportional to its binding affinity to liraglutide.

### Establishment of Animal Models

This study was ethically approved by the Institutional Animal Care and Use Committee (IACUC) of Peking University People's Hospital (No.2020PHE008). All procedures were performed in accordance with the guidelines of National Institutes of Health (NIH) Guide for the Care and Use of Laboratory Animals. 19

Humanized RBP4/TTR (normal) and RBP4/TTR^Val50Met^ (ATTRv) mice models were constructed in the Laboratory of Department of Endocrinology, Peking University People's Hospital and Beijing Cyagen Biological Science Co., LTD 20. The model construction procedures, which utilized CRISPR-Cas9 mRNA to knock in humanized RBP4/TTR or RBP4/TTRVal50Met genes into C57BL/6 zygotes followed by multi-generation breeding, were detailed in our previous research. For this study, 16-week-old male mice (range 13-19 weeks) with (Rbp4[KI/KI], Ttr[KI/KI]) and (Rbp4[KI/KI], Ttr[p.V50M][KI/KI]) genotypes were randomly assigned into two groups. Normal RBP4/TTR mice (n=6) received placebo treatment, while RBP4/TTR^Val50Met^ (ATTRv) mice were further randomized to receive either liraglutide (n=6) or placebo (n=6) treatment for a total of 28 days. Liraglutide was administered via intraperitoneal injection at a dose of 0.3mg/kg/day dissolved in normal saline, with a final solution volume of 10ml/kg/day based on the body weight of each mouse. When placebo administration was an intraperitoneal injection of equivalent dose of normal saline (10ml/kg/day). Day 0 (D0) was defined as the initiation day of the experiment, followed by daily administration until the completion at day 28 (week 4). Mice were housed at a constant temperature of 26℃ with a 12:12 hours light-dark cycle, with accommodations for 3-5 mice per cage. Unlimited water and fodders supplies were provided to each mouse. At the end of treatment (week 4), all the mice were sacrificed by cervical dislocation disposal. Cardiac, liver, and brain tissues were immediately excised and rapidly frozen in dry ice to preserve their integrity. The frozen tissues were then transferred and stored at -80°C to maintain their frozen state for subsequent analysis.

### Fasting plasma glucose and intraperitoneal glucose tolerance test (IPGTT)

Fasting plasma glucose and IPGTT were measured at D0, Week 2, and Week 4. Before the assay, mice should fast for 12 hours. Blood sample collection was implemented with the tail cutting methods. Fasting blood glucose level was tested priorly, and after acclimatizing for 30 minutes, an intraperitoneal injection of glucose (5% solution) was administered with the dose of 10ml/kg. Blood glucose measurements were repeated at the timing of 15 min, 30 min, 60 min, 90 min, and 120 min post-injection. Blood glucose was measured with automatic glucometer (Yuwell Medical Equipment Co., Ltd, China).

### Pathological staining

At week 4, cardiac specimens from each mouse were fixed with paraformaldehyde (PFA) followed by paraffin embedding. Cardiac tissues were then separated as right ventricle, ventricular septum, and left ventricle. Masson's trichrome staining was performed on the right ventricular myocardium to investigate ratio of collagen fiber deposition in the right ventricle, while hematoxylin-eosin (HE) staining was used for ventricular septum and left ventricle to measure the required parameters including ventricular septum thickness, left ventricular wall thickness, and left ventricular internal diameter. Brain tissue from mice was also fixed and paraffin embedded for pathological biopsy. Congo red staining was utilized to assess the percentage of amyloidosis in brain tissue sections. The area of Congo red-positive regions was measured as a percentage of the total section area using Image-Pro Plus 6.0 software (Media Cybernetics Inc., Rockville MD).

### Immunoblotting

At week 4, western blots were implemented to determine the protein levels of brain natriuretic peptide (BNP), transforming growth factor-beta (TGF-β), and collagen type I alpha 1 (COL1A1) in cardiac tissues (β-tubulin for internal reference), as well as the protein levels of TTR in liver and brain tissues (β-actin for internal reference). The samples were homogenized in lysis buffer (mixture of Tris-HCl pH 8.0, 50 mmol/L of sodium chloride, 1.0% NP-40, 0.5% sodium deoxycholate, 0.1% sodium dodecyl sulfate, and protease inhibitors), and the protein concentration was determined using a bicinchoninic acid (BCA) protein assay kit (Beyotime Biotechnology Co., Ltd, China) after homogenate centrifugation. To ensure accurate protein concentration measurement, a portion of the non-boiled samples (50 μL) was taken, boiled with loading buffer, and then measured. Following concentration determination, equal amounts of protein (50 μg) from each sample were adjusted and loaded onto SDS-PAGE gels.

For liver tissue samples, which were aimed at assessing TTR synthesis levels, proteins were denatured by heating in loading buffer at 95°C for 10 minutes. This denaturation step was necessary to linearize the proteins for accurate quantification of TTR expression. Non-reducing SDS-PAGE was performed without heat denaturation for brain tissue samples. This approach allowed us to differentiate between monomeric and multimeric forms of TTR, providing insights into the native state of TTR in the brain.

Equal amounts of 50 μg of protein were fractionated by sodium dodecyl sulfate-polyacrylamide gel electrophoresis (SDS-PAGE) (12% or 15% polyacrylamide gels). The gel was then transferred onto a polyvinylidene difluoride (PVDF) membrane using a transfer membrane apparatus (Millipore Trading Co., Ltd, USA) at 4 °C, applying a voltage of 100V/100mA for two hours. After blocking the membrane with tris buffered saline tween (TBST) blocking solution containing 5% skim milk powder, the samples were incubated overnight at 4 °C with the corresponding primary antibodies: BNP (Affinity Biosciences Co., Ltd, Australia), TGF-β (Huaan Bioscience Co., Ltd, China), COL1A1 (Abcam Trading Co., LTD., China), β-tubulin (Beijing Solarbio Science & Technology Co., Ltd, China), TTR (Abmart, China), and β-actin (Abmart, China). The membrane was then incubated with horseradish peroxidase (HRP) conjugated secondary antibodies (Beyotime Biotechnology Co., Ltd) (diluted with TBST blocking solution: Tris 10mM, NaCl 150mM, 0.05% Tween-20, pH 7.5) for 2 hours at 37 °C on a shaker.

To ensure equal protein loading across lanes, we used pre-stained molecular weight markers to monitor the transfer and to standardize the amount of protein loaded per lane. Detection of protein bands was achieved using a chemiluminescent substrate (ECL), and the signals were captured using a chemiluminescence imaging system (ImageQuant LAS 4000). The protein contents were measured with grey-scale scanning software (ImageJ 1.48, NIH) and were normalized to the corresponding internal references.

### Enzyme-linked immunosorbent (ELISA) assays

Serum BNP concentrations were measured by enzyme-linked immunosorbent assay (ELISA) at week 0, 2 and 4 after treatment. Blood sample was collected from the tail vein of mice. BNP was measured by mouse BNP kit (Wuhan Hualian Biotechnology Co., LTD., China), and all steps were performed in accordance with the instructions of each manufacturer.

### Real-time quantitative polymerase chain reaction (RT-qPCR)

The gene expression levels of *TTR*, *RBP4* in liver tissues were quantified using RT-qPCR. The RNA was extracted from liver tissues by Trizol (Invitrogen Trading Co., Ltd, USA) according to the protocols. RNA samples were reversely transcribed into cDNA thereafter and then into RT-qPCR amplification (Roche LightCycler® 480II, Roche Trading Co., Ltd, Switzerland) using the SYBR GREEN dye method. Specific primers for *TTR*, *RBP4*, and the housekeeping gene *HPRT* were designed to evaluate the mRNA levels. CT (cycle threshold) was defined as the minimum cycle turns for nucleic acid fluorescence signal to be detectable. Following the reaction, expression quantities of the above genes were calculated and rectified with *HPRT* as relative quantities through 2-ΔΔCT methods.

### TTR Stability Assay

TTR stability assay was performed to evaluate the influence of liraglutide on TTR tetramer stability. TTR monomers protein (3.6μM) were added to the experimental plate, when control plate was with equal quantities of normal saline. Both experimental and control plates were then incubated with liraglutide mixture (pH adjusted to 4.4) at 30°C for 25 minutes. The eventual concentrations of liraglutide were 0, 1.8, 3.6, 5.4 and 7.2 μM. The samples were further incubated at a temperature of 37°C for a period of 72 hours, when optical density was measured using UV-visible spectrometer at a wavelength of 245nm. The solution turbidity can indicate amyloid fibril formation. 21 In general, turbidity can be expressed as optical density, and turbidity is in correspondence with particular optical density at each wavelength. 22 Therefore, we calculated the difference in optical density between the experimental group and the control group at the same concentration of liraglutide to assess the depolymerization of TTR tetramer and the formation of TTR fibrils. An increase in optical density indicates an increase in the formation of TTR fibrils, which is a result of TTR depolymerization.

### Statistical analysis

The results of western blots were assessed and calculated through measurements performed using Image J software (NIH, Bethesda, MD, USA). Subsequently, data visualization was accomplished by creating graphs with Prism 8 software (GraphPad Software Inc., San Diego, CA, USA). Statistical analyses were implemented with IBM SPSS Version 26.0. For comparisons involving more than two groups, analysis of variance (ANOVA) was used, followed by Bonferroni's test for multiple comparisons. For comparisons between two groups, Student's t-test was used. Results were presented in the format of mean ± standard deviation (SD). Statistical significance was considered at P < 0.05.

## Results

In our study, humanized RBP4/TTR mice (mice with humanized transthyretin and retinol binding protein 4) and RBP4/TTR^Val50Met^ mice (mice with hereditary transthyretin amyloidosis) were established and divided into three groups by different genotypes and treatment strategies. A total of 18 mice were included and all experiment procedures were completed. Baseline characteristics were balanced among 3 treatment groups (**Table S1**).

### TTR interacted with liraglutide

**Figure 1:**
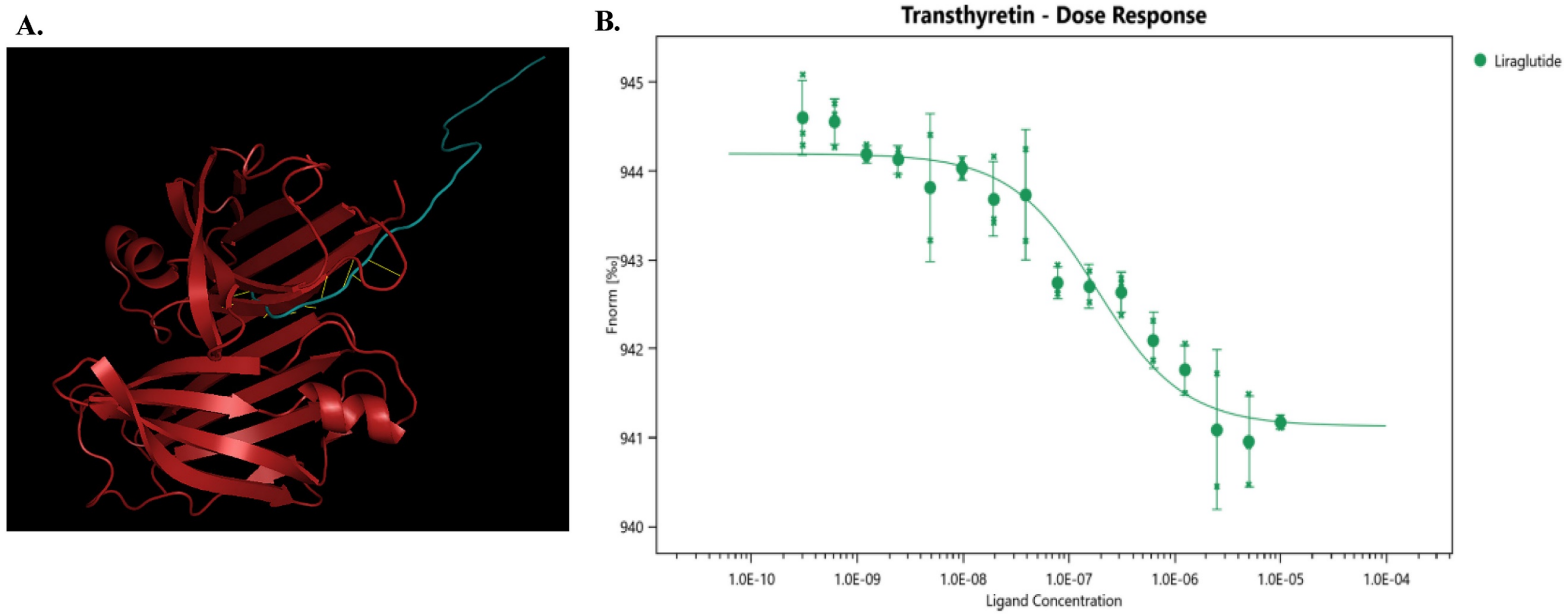
** (A)** The model diagram of liraglutide and TTR, made by Pymol. Red indicates TTR protein, blue indicates liraglutide, and yellow indicates mutual binding. This model aids in understanding how liraglutide interacts with TTR, offering insights into its potential therapeutic effects at the molecular level.** (B)** Liraglutide and TTR microscale thermography experiment. Kd(M): 1.569E-07 ± 6.37E-08; Signal to Noise: 10.7. The determined equilibrium dissociation constant (Kd) is 1.569E-07 ± 6.37E-08 M, indicating a strong binding affinity between liraglutide and TTR. The signal-to-noise ratio of 10.7 suggests high reliability of the experimental results.

High-throughput screening was used to identify potential targets of liraglutide, and TTR showed a ligand score of 2.98349 (**Figure 1A**), suggesting that TTR might be a promising target for liraglutide.

To further investigate the interaction between liraglutide and TTR, we conducted microscale thermophoresis experiments. With an increasing concentration of liraglutide, the TTR migration rate gradually increased, following an 'S'-shaped fitting curve. Moreover, the equilibrium dissociation constant (Kd) for liraglutide was 1.569E-07± 6.37E-08, indicating a direct combination between liraglutide and TTR (**Figure 1B**).

### Fasting glucose and intraperitoneal glucose tolerance test

**Figure 2:**
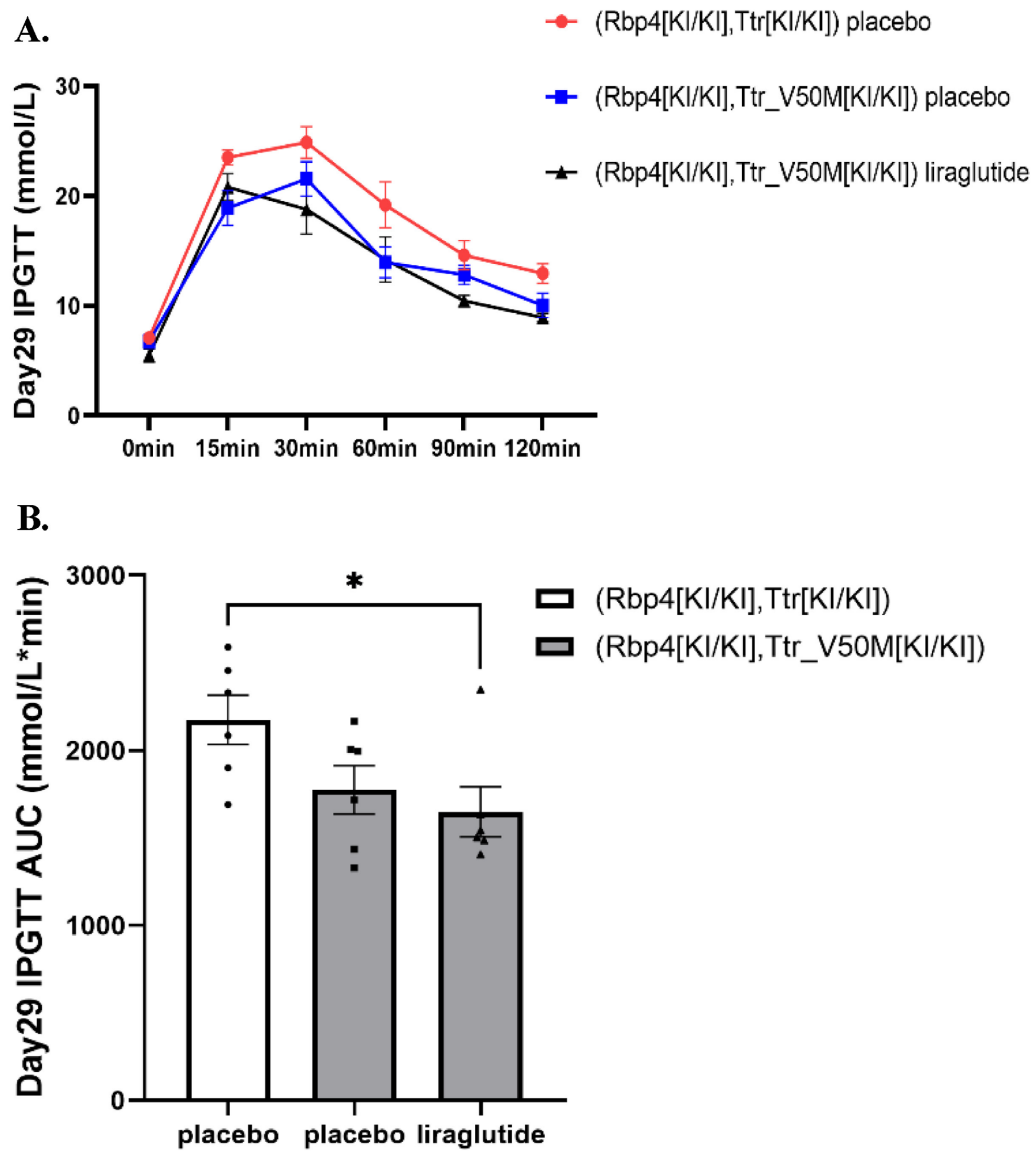
** (A)** The intraperitoneal glucose tolerance test (IPGTT) curve of mice on Day29 0-120min (n=6). The curve shows blood glucose levels at various time points, assessing glucose tolerance in mice.** (B)** Histogram of Day29 IPGTT area under the curve (AUC) data of mice (*: p<0.05; n=6). Statistically significant differences are marked with asterisks (: p<0.0.5), indicating significant variations in glucose tolerance among different treatment groups.

In ATTRv mice, no significant difference was found in fasting plasma glucose at week 0, 2, 4 among liraglutide and placebo groups (**Table S1**). On Day 29, the intraperitoneal glucose tolerance test (IPGTT) revealed a significant difference in glucose tolerance between the ATTRv placebo group and the RBP4/TTR liraglutide-treated group. As depicted in **Figure 2A**, the IPGTT curve for the ATTRv placebo group (red line) showed higher blood glucose levels across all time points compared to the RBP4/TTR liraglutide-treated group (black line). This was further confirmed by the area under the curve (AUC) analysis presented in **Figure 2B**, where the RBP4/TTR liraglutide-treated group exhibited a significantly lower AUC value (p < 0.05) compared to the ATTRv placebo group. These results indicate that liraglutide treatment significantly improves glucose tolerance in RBP4/TTR mice compared to the ATTRv placebo group. More relevant results were exhibited in **Figure 2A-2B** and **Table S1**.

### The effects of liraglutide on TTR monomers deposition and amyloidosis in mouse brain

**Figure 3:**
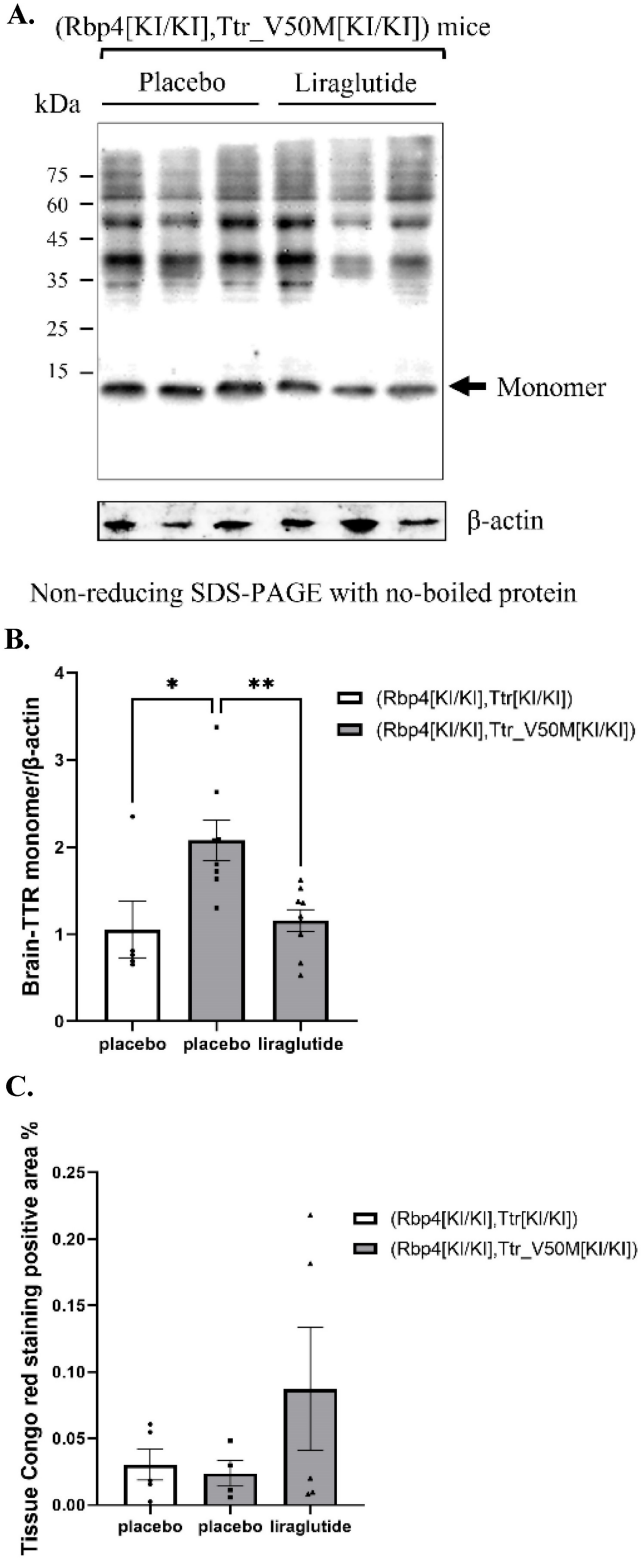
** (A)** Non-reducing SDS-PAGE of TTR monomer in mice brain (β-actin was boiled and used with SDS-PAGE). β-actin, boiled as an internal control, was used with SDS-PAGE to ensure consistent protein denaturation and quantification.** (B)** Results of TTR monomer protein expression in mice brain (*: p<0.05; **: p<0.01; n=6). Statistically significant differences are marked with asterisks (: p<0.05; **: p<0.01), indicating significant variations in TTR monomer expression among different treatment groups.** (C)** Congo Red positive area percentage of mouse brain tissue. Each experiment was repeated three times (n=6). Congo Red staining detects amyloid protein deposits, and the percentage of positive area reflects the severity of amyloidosis.

It was indicated that in mice with placebo treatment, the TTR protein levels in the brain tissue of ATTRv mice increased compared with humanized RBP4/TTR mice (p=0.014). In addition, liraglutide treatment in ATTRv mice led to a reduction in the monomeric TTR protein levels in brain tissue, as compared to placebo (p=0.009) (**Figure 3A-B**). While for following Congo red staining of brain tissue, there was no notable variance observed in the area affected by amyloidosis across all treatment groups (**Table S1** and** Figure 3C**).

### The effects of liraglutide on cardiac amyloidosis and heart failure in mice

**Figure 4:**
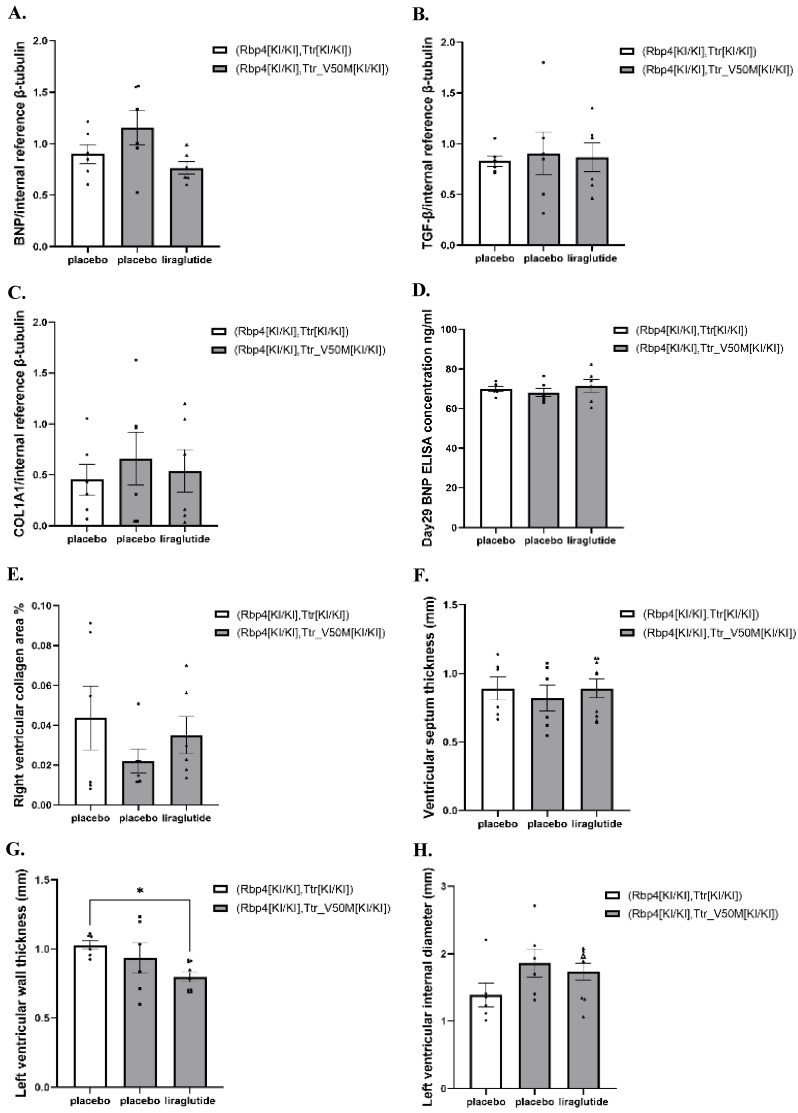
** (A)** Results of BNP protein expression in mice heart (n=6).** (B)** Results of TGF-β protein expression in mice heart (n=6).** (C)** Results of COL1A protein expression in mice heart (n=6).** (D)** Results of Day29 BNP concentration in mice serum (n=6).** (E)** Results of collagen fiber deposition area in the right ventricle of mice (n=6).** (F)** Results of ventricular septum thickness (n=6).** (G)** Results of left ventricular wall thickness (n=6).** (H)** Results of left ventricular internal diameter (n=6). These results assess the effects of liraglutide on cardiac function and structure.

In both normal and ATTRv mice, at week 4, the protein levels of BNP, TGF-β, and COL1A1 in the cardiac tissues did not exhibit significant changes (**Table S1**,** Figure 4A-C** and **Figure S1-S2**). Meanwhile, at week 4, in both normal and ATTRv mice, the plasma BNP concentrations were comparable among all treatment groups (**Table S1** and** Figure 4D**). In addition, the pathological inspection indicated that the right ventricular collagen fiber deposition area, interventricular septum thickness, left ventricular wall thickness, and left ventricular diameter were comparable among ATTRv mice & liraglutide, ATTRv mice & placebo, and normal mice & placebo groups (**Table S1** and** Figure 4E-H**).

### The effects of liraglutide on synthesis of TTR and RBP4 in mouse liver

**Figure 5:**
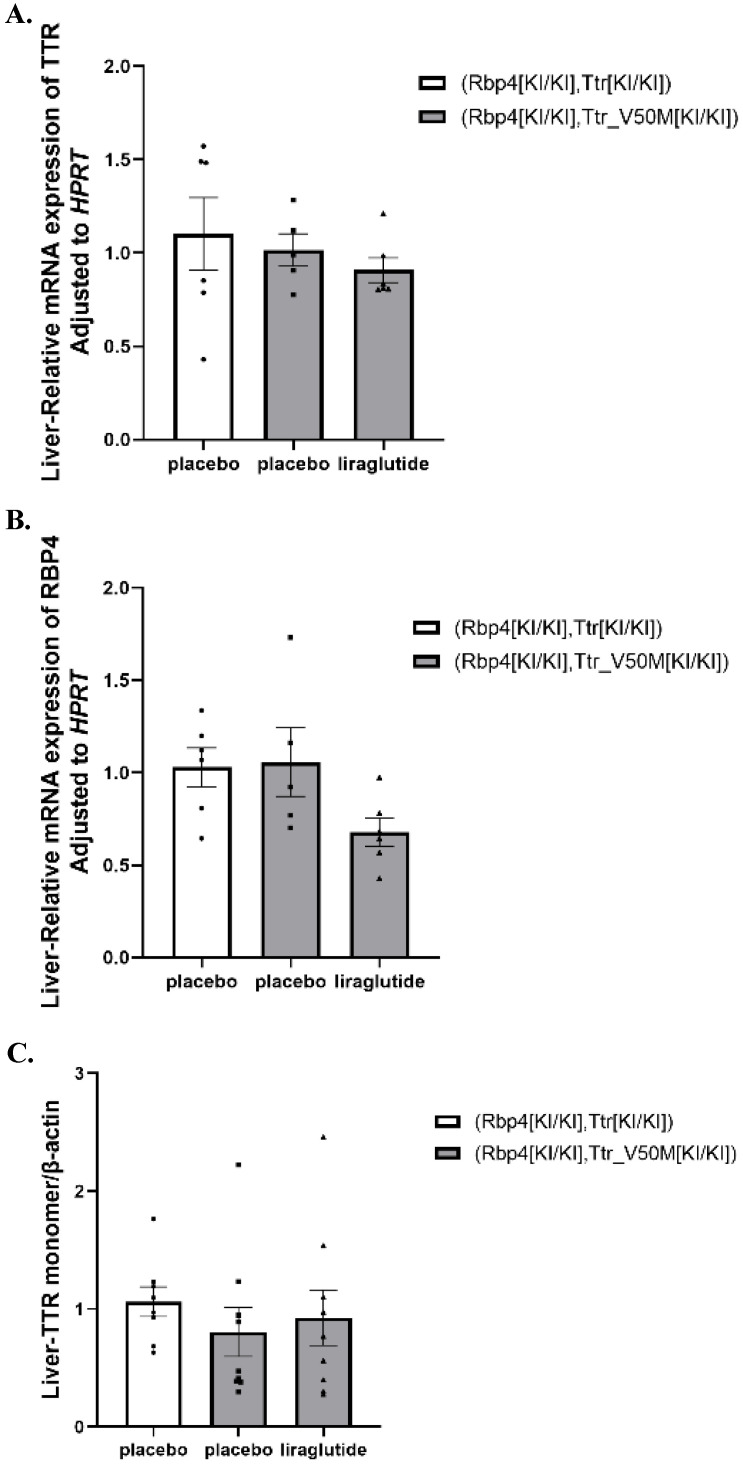
** (A)** Results of TTR mRNA expression in mice liver (n=6).** (B)** Results of RBP4 mRNA expression in mice liver (n=6).** (C)** Results of TTR monomer protein expression in mice liver (n=6). These results evaluate the effects of liraglutide on liver TTR and RBP4 synthesis. These results evaluate the effects of liraglutide on liver TTR and RBP4 synthesis.

No significant difference in the gene expression of *TTR*, *RBP4*, and the protein levels of TTR in the liver among all 3 treatment groups were identified (**Table S1**,** Figure 5A-C** and **Figure S3-S4**).

### The effects of liraglutide on TTR stability

**Figure 6:**
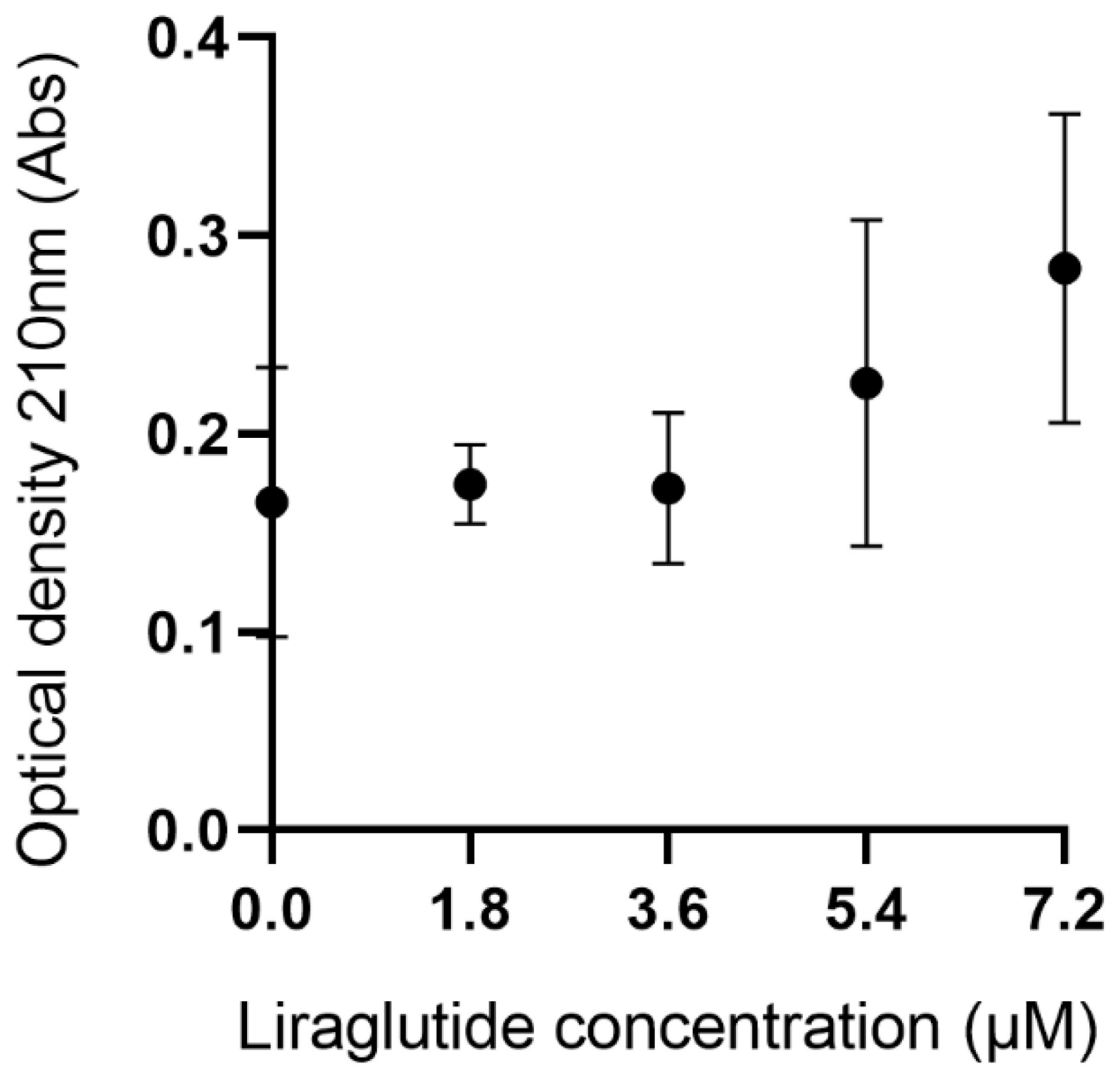
Results of TTR stability experiment. The experiment measures changes in optical density of TTR tetramers in the presence of various concentrations of liraglutide, assessing the impact of liraglutide on TTR stability.

To assess the effect of liraglutide on TTR stability, we measured the optical density (OD) at 210 nm of TTR tetramers in the presence of various concentrations of liraglutide (0.0, 1.8, 3.6, 5.4, and 7.2 μM). As shown in **Figure 6**, the mean OD values were 0.166 at 0.0 μM, 0.175 at 1.8 μM, 0.173 at 3.6 μM, 0.226 at 5.4 μM, and 0.284 at 7.2 μM. Statistical analysis revealed no significant differences in TTR stability across the tested liraglutide concentrations (p > 0.05), indicating that the stability of TTR tetramers was not significantly influenced by the increase in liraglutide concentration.

## Discussion

As far as we know, this research utilized liraglutide in ATTR animal models for the first time, which is an emerging attempt to investigate the protective effects and underlying mechanisms of liraglutide in cardiovascular and nervous conditions raised by ATTR, and is a novel exploration for drug therapy in ATTR.

In this study, we found that TTR was a potential target of liraglutide, and successfully demonstrated that TTR had high affinity for liraglutide with direct binding. These discoveries provided novel insights for the potential mechanisms of liraglutide remedying ATTR, and would promote further pre-clinical and clinical investigations on this topic.

As mentioned above, liraglutide exhibited protective effects on several neuro-degenerative diseases. 10 Studies have found that liraglutide can enhance the *Wnt/β-catenin* signaling pathway in mouse brain tissue, then promote the expression of neurogenic markers and anti-apoptotic factors, and improve cognitive impairment. 23 In addition, liraglutide can also reduce the oxidative stress of neuronal mitochondria, then reduce the neuronal inflammatory response, and play a role in brain injury repair. 24,25 However, for ATTR-related central neural diseases (brain amyloidosis and degenerative lesions), no previous research evaluating the therapeutic potential of liraglutide has been conducted, and whether liraglutide would improve the prognosis in patients with ATTR related remains unclear. According to the results, we found a decrease in TTR monomer contents in ATTRv mice under liraglutide treatment compared with placebo, indicating improvement of brain TTR monomer deposition in protein levels. However, no significant decrease in amyloidosis area of ATTRv mice brain tissue were observed by Congo red stained sections, meaning that the evidence of brain amyloidosis improvement in histological levels remains lacking. Previous studies have identified nonfibrillar, Congo Red-negative transthyretin aggregates in early-stage familial amyloid polyneuropathy, indicating the presence of preamyloidogenic TTR species in nerves before fibril formation, whereas mature fibrils do not exhibit such cytotoxicity 26-28. And this research suggests that the accumulation of nonfibrillar, Congo Red-negative TTR cytotoxic deposits—below the detectability threshold of Congo Red—may underlie early neuropathology 26-28.

This is further supported by evidence that prealbumin (TTR) is a common component of neurotic plaques in cerebral amyloidosis and that nonfibrillar, Congo Red-negative preamyloidogenic TTR species in nerves induce cytotoxic effects (including oxidative stress) in both animal models and human studies prior to the appearance of congophilic fibrillar amyloid 29-32. Notably, it has also been suggested that amyloidosis area in pathological sections does not fully reflect the progression and severity of ATTR-PN. 33 Our research results aligned with these observations. Since liraglutide exhibited direct combining ability to TTR, it might improve the overall neural prognosis in patients with ATTR. More studies examining the specific effects of liraglutide on the pathological changes and neurological function in patients with ATTR are warranted in the future.

Notably, while the present study explored liraglutide's potential in modulating TTR metabolism for central nervous system pathologies, its broader therapeutic profile as a GLP-1RA—which has been extensively linked to cardiovascular protection via mechanisms such as anti-inflammation, endothelial function improvement, and atherosclerotic plaque stabilization—raises questions about whether its systemic effects on receptor signaling could underlie benefits across diverse organ systems, including both neural and cardiovascular tissues.

Previous research indicates that GLP-1RAs offer substantial cardiovascular advantages for individuals with or without T2DM, primarily by lowering the incidence of MACE (major adverse cardiovascular events) rates, which encompasses MI (myocardial infarction), stroke, and cardiovascular death. 34 Among the eight CVOTs (cardiovascular outcome trials) conducted on T2DM patients, five have reported positive outcomes 35-39, while three indicate neutral cardiovascular effects. 40-42 The HARMONY trial is particularly noteworthy as it implies that the cardiovascular benefits of GLP-1RAs might not be contingent upon weight reduction. 36

GLP-1RAs also hold promise in the management of heart failure, especially HFpEF (heart failure with preserved ejection fraction), a condition prevalent among obese individuals and those with T2DM. 43 A meta-analysis encompassing over 60,000 T2DM patients from GLP-1RA CVOTs has revealed an 11% reduction in heart failure-related hospitalizations. 34 In a specific trial, semaglutide was found to alleviate HF-related symptoms and enhance exercise capacity in individuals with obesity, HFpEF, and T2DM. 44

In the context of coronary artery disease, a pooled analysis of GLP-1RA CVOTs involving T2DM patients has indicated a 10% decrease in nonfatal MI. 34 This suggests that GLP-1RAs may possess anti-atherogenic properties that could influence various pathways associated with plaque development and progression.^45^

The SCALE Diabetes program and STEP 2 trial both have revealed that semaglutide enables a significant proportion of participants to achieve prediabetic HbA1c levels and improves various cardiovascular risk factors. 46,47 As a result, major diabetes associations have updated their guidelines to recommend GLP-1RAs for T2DM patients with established cardiovascular disease (CVD) or high-risk indicators, aiming to reduce the risk of MACE. 48-50

The efficacy of GLP-1RA in improving cardiometabolic health in patients living with T2DM has spurred significant efforts toward developing next-generation therapies that surpass the effectiveness of GLP-1RA alone. 51 Novel dual agonists (targeting GLP-1R/GIPR such as tirzepatide) and triple agonists (targeting GLP-1R/GIP/GCG such as retatrutide) have demonstrated enhanced effects on weight loss and metabolic health outcomes in obesity, T2DM, and CVD. 52 In the SURPASS trials, tirzepatide has showed more pronounced reductions in TG, LDL-C, VLDL-C, and systolic blood pressure compared to semaglutide, while also lowering inflammatory markers in a Phase 2 trial. 53-57 Retatrutide, in a Phase 2 trial, has showed a dose-dependent decrease in systolic blood pressure. 58 Ongoing trials like SURPASS-CVOT and TRIUMPH-2/3 aim to systematically evaluate the safety profiles and cardiometabolic benefits of these agonists in T2DM and CVD populations. 59 The primary endpoints will focus on time to first occurrence of MACE and other relevant clinical outcomes, helping clarify whether their benefits arise from enhanced weight loss or direct receptor-mediated effects on metabolic organs. 59-61

As clinical evidence robustly supports the cardiometabolic benefits of GLP-1RAs across diverse patient populations, their mechanistic basis, which encompasses both systemic metabolic improvements and direct cellular interactions, has become a focal point of research. GLP-1RAs mediate their effects through the GLP-1 (glucagon-like peptide 1) class B G protein-coupled receptor, which is broadly expressed in metabolic tissues such as pancreatic β-cells and cardiovascular structures including cardiac myocytes and vascular smooth muscle cells. 62-65 The cardiovascular advantages of GLP-1RAs may derive from dual pathways: indirect benefits via improved glycemic control and weight management in type 2 diabetes patients, and direct interactions with the cardiovascular system, supported by GLP-1 receptor expression in cardiac and vascular cell types. 64,65 In preclinical investigations using atherosclerosis-susceptible mouse models, GLP-1RAs such as semaglutide and liraglutide have been shown to mitigate atherosclerotic plaque progression independent of weight loss or cholesterol reduction, potentially through anti-inflammatory mechanisms like inhibition of Toll-like receptor-induced inflammation. 66-68 Furthermore, in nondiabetic hypertensive mice, liraglutide normalizes blood pressure, reduces cardiac hypertrophy, vascular fibrosis, and endothelial dysfunction in a GLP-1 receptor-dependent manner, while protecting against myocardial ischemia-reperfusion injury via endothelial GLP-1 receptor signaling; this was validated by blunted cardioprotective effects in mice with endothelial-specific ablation of the GLP-1 receptor gene. 65, 69-71

Though preclinical and clinical evidence strongly supports GLP-1RA-mediated cardiovascular protection through multi-mechanistic pathways in diverse models, the specific effects of liraglutide on cardiovascular endpoints in TTR-related cardiac pathologies including myocardial fibrosis and heart failure remain underexplored. In our study, several correlative indexes were also measured to evaluate the cardiovascular outcomes in mice with liraglutide treatment. It is well known that TGF-β serves as a major cytokine whose activation aggravates cardiac fibrosis and hypertrophy. 26 Similarly, COL1A1 promotes pro-collagen synthesis and collagen fibers formation extracellularly, which could also be used as an indicator for cardiac fibrosis. 27 Meanwhile, BNP as a recognized marker, is also an authentic measurement for degrees of heart failure 28,72, and pathological sections can be used to observe the presence of cardiac mal-remodeling. Therefore, we observed the contents of TGF-β, COL1A1, BNP proteins in heart tissues, and investigated pathological sections of the cardiac of mice to observe the therapeutic effect of liraglutide in respect to myocardial fibrosis and heart failure. Serum BNP was also measured for evaluation of heart failure progression and severity. However, the results revealed no significant difference in the degree of cardiac fibrosis and the severity of heart failure after liraglutide treatment. This observation might be attributed to the relatively short observation period in our study, which could have been insufficient to detect relevant cardiovascular outcomes in the mice. Therefore, further researches investigating the efficacy of liraglutide in ATTR-CM with longer study periods and more outcome evaluating aspects are still required.

Moreover, TTR proteins are mainly synthesized in the liver, and exendin-4 (a GLP-1 analogue) was elucidated with the ability to influence the expression of multiple microRNAs in liver cells and thereby correspondingly adjust hepatic metabolism and biological synthesis 73. In order to investigate whether liraglutide affects the synthesis process of TTR, we conducted several in-depth investigations. The results showed that compared with placebo, the effects of liraglutide on liver TTR synthesis in ATTRv mice did not reach a statistical difference, and the regulatory role of GLP-1RA in TTR synthesis of liver needs further research.

The instability of TTR tetramer was also thought to be a key step in the formation of ATTR related amyloidosis, which has promoted the discovery and development of TTR tetramer stabilizers. The clinical efficacy of TTR tetramer stabilizers diflunisal and tafamidis in treatment of ATTR has been confirmed in previous studies 1,74, and we therefore further explored whether liraglutide could similarly influence the stability of TTR tetramers. By stability assay, we observed comparable optical density of TTR tetramers solution before and after incubation with liraglutide, which indicated that liraglutide might not significantly influence the stability of TTR tetramers. While since TTR tetramers showed high affinity and direct bond to liraglutide, further researches elucidating the underlying biological effects of their combination should be carried out.

Our study presents several limitations that should be considered when interpreting the findings. Firstly, the use of only male mice in our experiments precludes the assessment of potential gender differences in response to liraglutide treatment. Future studies should include both male and female mice to provide a more comprehensive understanding of the drug's effects across sexes. Secondly, since the dose or duration of liraglutide treatment may not be optimal for observing beneficial effects in this model, the results should be interpreted with caution, and additional researches with varying treatment regimens are warranted. Thirdly, the observation period in our study was insufficient to detect relevant cardiovascular outcomes in the mice, which may have contributed to the lack of significant findings in cardiac fibrosis and heart failure severity. Meanwhile, since the findings in this study were primarily negative, we did not implement subsequent explorations of promising downstream pathways involved in liraglutide for TTR deposition. Nevertheless, as the brain TTR contents were reduced after liraglutide treatment in ATTR mice models, it is meaningful to further investigate the underlying pathways. More researches regarding the potential mechanisms for GLP-1RA improving ATTR are still warranted.

Despite these limitations, our study provides valuable insights as the first investigation into the effects of liraglutide on brain and cardiovascular conditions associated with ATTRv. The findings highlight the need for further research to validate our observations and to explore the potential efficacy and safety of GLP-1RAs in the treatment of ATTR.

## Conclusion

In this study, TTR was found to be a potential target of liraglutide, and the direct interaction between them was also observed. In ATTRv mice, liraglutide significantly reduced the contents of TTR monomers in brain tissues compared with placebo, while the specific benefits towards neural function or pathological changes warrant further exploration. However, there was no evidence that liraglutide improved the cardiovascular prognosis of mice with ATTRv. More researches to validate these findings and demonstrate the underlying mechanisms are still required.

## Supplementary Material

Supplementary figures and tables.
