# Supplementary material for: Cardiovascular and brain effects of liraglutide in transthyretin amyloidosis (ATTR) mice models
Source: Int J Med Sci. 2025 Jul 10;22(13):3229–41. doi: 10.7150/ijms.112264 (PMC12320650; doi:10.7150/ijms.112264)
Supplement: Supplementary file 1 — Supplementary figures and tables. [file ijmsv22p3229s1.pdf]

## Supplementary materials

**Supplementary figures**

Figure S1. The protein expression of BNP, TGF-β and COL1A in the cardiac of (Rbp4[KI/KI],Ttr [KI/KI]) mice.

Figure S2. The protein expression of BNP, TGF-β and COL1A in the cardiac of (Rbp4[KI/KI],Ttr_V50M[KI/KI]) mice.

Figure S3. The protein expression of TTR monomer in the liver of (Rbp4[KI/KI],Ttr [KI/KI]) mice.

Figure S4. The protein expression of TTR monomer in the liver of (Rbp4[KI/KI],Ttr_V50M[KI/KI]) mice.

**Supplementary tables**

Table S1. Summarization of study results regarding different outcomes.

Figure S1. The protein levels of BNP, TGF-β and COL1A in (Rbp4[KI/KI],Ttr [KI/KI]) mice heart.

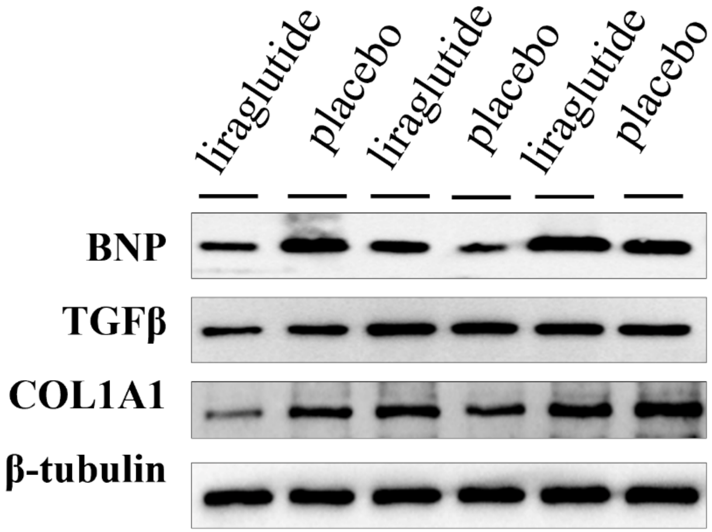


Figure legends: Western blots characterizing the protein levels of BNP, TGF-β and COL1A in the cardiac of (Rbp4[KI/KI],Ttr [KI/KI]) mice.

Figure S2. The protein levels of BNP, TGF-β and COL1A in the cardiac of (Rbp4[KI/KI],Ttr_V50M[KI/KI]) mice.

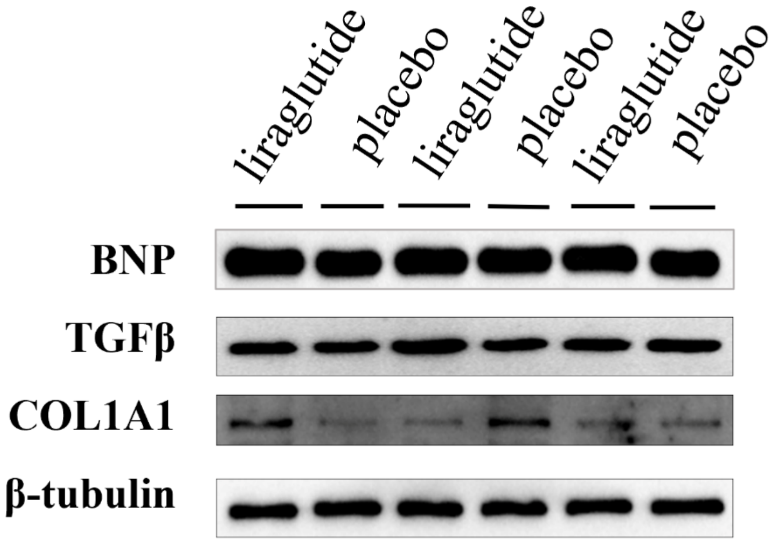


Figure legends: Western blots characterizing the protein levels of BNP, TGF-β and COL1A in the cardiac of (Rbp4[KI/KI],Ttr_V50M[KI/KI]) mice.

Figure S3. The protein levels of TTR monomer in the liver of (Rbp4[KI/KI],Ttr [KI/KI]) mice.

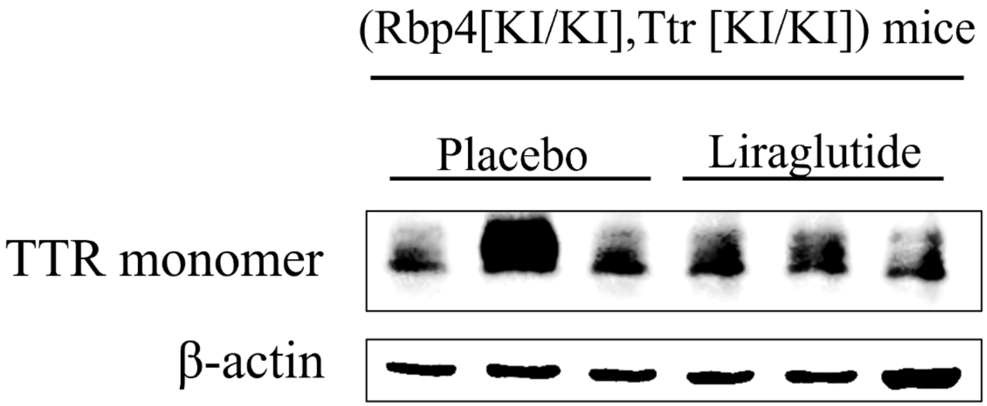


Figure legends: Western blots characterizing the protein levels of TTR monomer in the liver of (Rbp4[KI/KI],Ttr [KI/KI]) mice.

Figure S4. The protein expression of TTR monomer in the liver of (Rbp4[KI/KI],Ttr V50M[KI/KI]) mice.

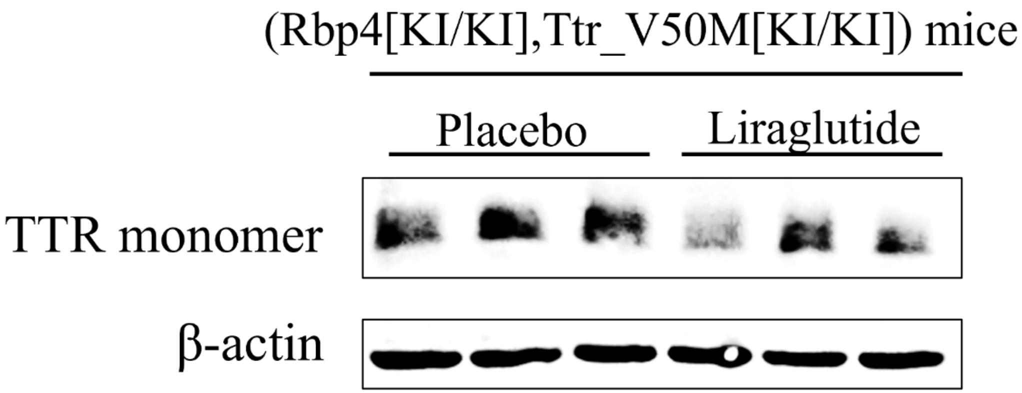


Figure legends: Western blots characterizing the protein levels of TTR monomer in the liver of (Rbp4[KI/KI],Ttr V50M[KI/KI]) mice.

Table S1. Summarization of study results regarding different outcomes

| **Outcome measurements** | **Liraglutide treated arms** | **Placebo treated arms** | **P values** |
|---|---|---|---|
| Liraglutide treatment versus placebo treatment in mice with (Rbp4[KI/KI],Ttr[p.V50M][KI/KI]) genotype | | | |
| Weight D0 (g) | 24.77 ± 2.06 | 25.23 ± 1.48 | 0.66 |
| Weight D9 | 26.07 ± 2.78 | 27.10 ± 2.56 | 0.52 |
| Weight D16 | 26.38 ± 2.04 | 27.40 ± 2.89 | 0.50 |
| Weight D23 | 26.95 ± 2.10 | 27.58 ± 2.37 | 0.64 |
| Weight D29 | 24.68 ± 2.05 | 25.50 ± 2.10 | 0.51 |
| Weight change from baseline (D16) | 1.62 ± 1.35 | 2.17 ± 2.70 | 0.67 |
| Weight change from baseline (D29) | -0.083 ± 1.59 | 0.27 ± 1.72 | 0.72 |
| Fasting blood glucose D0 (mmol/L) | 6.65 ± 1.19 | 6.57 ± 0.50 | 0.88 |
| Fasting blood glucose D16 | 6.55 ± 2.74 | 6.47 ± 0.88 | 0.95 |
| Fasting blood glucose D29 | 5.48 ± 1.49 | 6.77 ± 1.51 | 0.17 |
| **IPGTT AUC D0 (mmol/L*min)** | **1778.50 ± 156.14** | **1603.75 ± 109.88** | **0.049** |
| IPGTT AUC D16 | 1253.00 ± 361.49 | 1402.75 ± 281.87 | 0.44 |
| IPGTT AUC D29 | 1649.75 ± 348.22 | 1773.88 ± 338.76 | 0.55 |
| Wb BNP/β tubulin | 0.76 ± 0.15 | 1.16 ± 0.40 | 0.05 |

| | | | |
|---|---|---|---|
| Wb TGF-β/β tubulin | 0.87 ± 0.35 | 0.90 ± 0.52 | 0.88 |
| Wb COL1A1/β tubulin | 0.54 ± 0.51 | 0.66 ± 0.63 | 0.73 |
| ELISA BNP concentration D0 (ng/ml) | 74.28 ± 5.90 | 76.61 ± 1.98 | 0.38 |
| ELISA BNP concentration D16 | 65.58 ± 5.85 | 64.19 ± 5.26 | 0.67 |
| ELISA BNP concentration D29 | 71.29 ± 8.17 | 68.09 ± 5.07 | 0.43 |
| Right ventricular collagen percentage (%) | 0.035 ± 0.023 | 0.022 ± 0.015 | 0.70 |
| Ventricular septum thickness (µm) | 869.95 ± 187.52 | 800.96 ± 154.01 | 0.81 |
| Left ventricular wall thickness (µm) | 807.95 ± 123.05 | 951.43 ± 221.90 | 0.25 |
| Left ventricular internal diameter (µm) | 1553.53 ± 353.08 | 1773.54 ± 151.66 | 0.48 |
| **Outcome measurements** | **(Rbp4[KI/KI], Ttr[KI/KI]) genotype** | **(Rbp4[KI/KI],Ttr[p.V50M][KI/KI]) genotype** | **P values** |
| (Rbp4[KI/KI], Ttr[KI/KI]) versus (Rbp4[KI/KI],Ttr[p.V50M][KI/KI]) in mice treated with placebo | | | |
| Weight D0 (g) | 24.45 ± 3.16 | 25.23 ± 1.48 | 0.60 |
| Weight D9 | 26.23 ± 2.78 | 27.10 ± 2.56 | 0.59 |
| Weight D16 | 25.75 ± 2.39 | 27.40 ± 2.89 | 0.31 |
| Weight D23 | 25.68 ± 2.16 | 27.58 ± 2.37 | 0.18 |
| Weight D29 | 24.30 ± 2.89 | 25.50 ± 2.10 | 0.43 |
| Weight change from baseline (D16) | 1.30 ± 1.58 | 2.17 ± 2.70 | 0.51 |

| | | | |
|---|---|---|---|
| Weight change from baseline (D29) | -0.15 ± 1.53 | 0.27 ± 1.72 | 0.67 |
| Fasting blood glucose D0 (mmol/L) | 7.40 ± 1.72 | 6.57 ± 0.50 | 0.28 |
| Fasting blood glucose D16 | 7.25 ± 1.98 | 6.47 ± 0.88 | 0.40 |
| Fasting blood glucose D29 | 7.08 ± 1.33 | 6.77 ± 1.51 | 0.71 |
| IPGTT AUC D0 (mmol/L*min) | 1785.00 ± 373.89 | 1603.75 ± 109.88 | 0.28 |
| IPGTT AUC D16 | 1641.38 ± 296.52 | 1402.75 ± 281.87 | 0.18 |
| IPGTT AUC D29 | 2174.50 ± 344.85 | 1773.88 ± 338.76 | 0.07 |
| Wb BNP/β tubulin | 0.90 ± 0.23 | 1.16 ± 0.40 | 0.21 |
| Wb TGF-β/β tubulin | 0.83 ± 0.13 | 0.90 ± 0.52 | 0.74 |
| Wb COL1A1/β tubulin | 0.45 ± 0.37 | 0.66 ± 0.63 | 0.50 |
| ELISA BNP concentration D0 (ng/ml) | 74.74 ± 4.42 | 76.61 ± 1.98 | 0.37 |
| ELISA BNP concentration D16 | 66.12 ± 3.91 | 64.19 ± 5.26 | 0.49 |
| ELISA BNP concentration D29 | 70.01 ± 3.04 | 68.09 ± 5.07 | 0.44 |
| Right ventricular collagen percentage (%) | 0.044 ± 0.039 | 0.022 ± 0.015 | 0.39 |
| Ventricular septum thickness (μm) | 906.85 ± 324.15 | 800.96 ± 154.01 | 0.84 |
| Left ventricular wall thickness (μm) | 1013.48 ± 69.83 | 951.43 ± 221.90 | 0.58 |
| Left ventricular internal diameter (μm) | 1546.00 ± 816.62 | 1773.54 ± 151.66 | 0.84 |
